# Osseointegration at Implants Installed in Composite Bone: A Randomized Clinical Trial on Sinus Floor Elevation

**DOI:** 10.3390/jfb13010022

**Published:** 2022-02-28

**Authors:** Mitsuo Kotsu, Karol Alí Apaza Alccayhuaman, Mauro Ferri, Giovanna Iezzi, Adriano Piattelli, Natalia Fortich Mesa, Daniele Botticelli

**Affiliations:** 1 ARDEC Academy, 47923 Rimini, Italy; dental_rescue@yahoo.co.jp; 2 Department of Oral Biology, Medical University of Vienna, 1090 Vienna, Austria; caroline7_k@hotmail.com; 3 ARDEC Foundation, Cartagena de Indias 130001, Colombia; medicina2000ctg@hotmail.com; 4 Department of Medical Oral and Biotechnological Sciences, University of Chieti-Pescara, 66100 Chieti, Italy; gio.iezzi@unich.it (G.I.); apiattelli@unich.it (A.P.); 5 School of Dentistry, University Corporation Rafael Núñez, Cartagena de Indias 130001, Colombia; natalia.fortich@curnvirtual.edu.co

**Keywords:** maxillary sinus, biomaterial, sinus augmentation, collagen membrane, access window, antrostomy, osteotomy

## Abstract

Osseointegration of implants installed in conjunction with sinus floor elevation might be affected by the presence of residual graft. The implant surface characteristics and the protection of the access window using a collagen membrane might influence the osseointegration. To evaluate these factors, sinus floor elevation was performed in patients using a natural bovine bone grafting material. The access windows were either covered with a collagen membrane made of porcine corium (Mb group) or left uncovered (No-Mb group) and, after six months, two mini-implants with either a moderate rough or turned surfaces were installed. After 3 months, biopsies containing the mini-implants were retrieved, processed histologically, and analyzed. Twenty patients, ten in each group, were included in the study. The two mini-implants were retrieved from fourteen patients, six belonging to the Mb group, and eight to the No-Mb group. No statistically significant differences were found in osseointegration between groups. However, statistically significant differences were found between the two surfaces. It was concluded that implants with a moderately rough surface installed in a composite bone presented much higher osseointegration compared to those with a turned surface. The present study failed to show an effect of the use of a collagen membrane on the access window.

## 1. Introduction

Sinus floor elevation through a lateral access is a well-documented procedure used to increase bone volume in the posterior segments of the maxilla [1]. This approach includes the elevation of the sinus mucosa and the immediate placement of biomaterial [2,3], devices [4,5,6], implants alone [7,8], or in conjunction with biomaterial [9], aiming to maintain over time the elevated volume and allow bone growth within the subantral space [10,11,12,13,14]. The use of a membrane to cover the lateral bone window has been suggested to improve implant success [1] and might decrease both the dislodgment of the biomaterial through the access window [15,16] and the post-surgical morbidity [16]. Nevertheless, a systematic review with meta-analysis [17] failed to find effects on bone formation placing a membrane on the access window.

The implant surface instead might influence osseointegration. In an experimental study in dogs in which the osseointegration of a moderately rough surface was compared with a turned surface, better outcomes were observed at the former compared to the latter surface [18]. Even though good long-term results can be achieved also with turned surfaces [19], in a systematic review it was concluded that the best survival rate of implants installed in combination with sinus floor elevation was obtained by implants with a rough surface [1].

Nevertheless, experimental studies showed a higher progression of peri-implantitis at rough compared to turned surfaces [20]. However, systematic reviews concluded that the surface did not seem to affect the incidence of peri-implantitis [21,22]. In a retrospective study in patients with a history of periodontitis, a hybrid surface, i.e., presenting a turned surface limited to the coronal part and the remaining portion of the implant with a rough surface, showed less marginal bone loss compared to a conventional rough surface [23]. However, no clinical, radiographic, and microbiological differences were found between hybrid and traditional implants in a randomized clinical trial (RCT) in patients with history of periodontitis [24]. Even though a turned surface presented high clinical results when installed in pristine alveolar bone [19], the conditions for osseointegration might be compromised by the presence of regenerated composite bone, composed of newly formed bone and residual graft particles. In a human study after sinus floor elevation, biopsies taken from the elevated regions and from pristine zones were evaluated [25]. Both groups presented ~46% of vital bone. It has to be considered that immediate and delayed implants present different behaviors after installation in composite bone. In an experimental study in dogs, circumferential marginal defects were immediately filled with deproteinized bovine bone matrix [26]. Only few particles were found in contact to the implant surface after 4 months of healing. Instead, at implants installed after 6 months of healing after sinus floor elevation, up to ~16% of the implant surface was found in contact to graft particles, reducing the space available for osseointegration by up to 32% [27,28]. It should be considered that human biopsies harvested from the distal segments of the maxilla after 6 weeks of healing resulted in ~46–47% of osseointegration [29]. It might be argued that in a delayed mode the graft particles are stuck into newly formed bone, so that osteotomy preparation and implants might impact with the graft. Instead, when an immediate mode is applied, new bone has the chance to be formed between the implant and graft surfaces separating the particles from the implant.

Under such conditions, implant surface quality and osteoconductivity might acquire great importance.

Hence, the aim of the present study was to evaluate the osseointegration of different surfaces installed into composite bone. Moreover, the influence of the use of a collagen membrane on the access window was also assessed.

## 2. Materials and Methods

### 2.1. Ethical Statement

The protocol of the present randomized controlled trial (RCT) was approved by the Ethical Committee of the University Corporation Rafael Núñez, Cartagena de Indias, Colombia (protocol #02-2015; 19 May 2015). The study was carried out at the same university. The Declaration of Helsinki on medical protocols and ethics were adopted. All participants signed informed consent after being thoroughly notified about procedures and possible complications. The CONSORT checklist was followed to structure the article. The present study reports the histological finding while, in a previous RCT article, tomographic evaluations of the dimensional changes of the augmented space after sinus floor elevation were reported [30]. The RCT was registered at ClinicalTrials.gov with the following identifier code: NCT03899688.

### 2.2. Study Population

The inclusion criteria were the following: (i) the presence of an edentulous zone in the posterior segment of the maxilla presenting a height of the sinus floor ≤4 mm; (ii) requesting a fix prosthetic rehabilitation on implants in that region; (iii) ≥21 years of age; (iv); and (V) not being pregnant. The following excluding criteria were adopted: (i) no contraindications for oral surgical procedures; (ii) under chemotherapic or radiotherapeutic treatment; (iii) presence of an acute or a chronic sinusitis; and (iv) previous bone augmentation procedures in the region. Smokers of >10 cigarettes per day and patients under bisphosphonates treatment were also excluded.

### 2.3. Devices and Biomaterials

**Figure 1:**
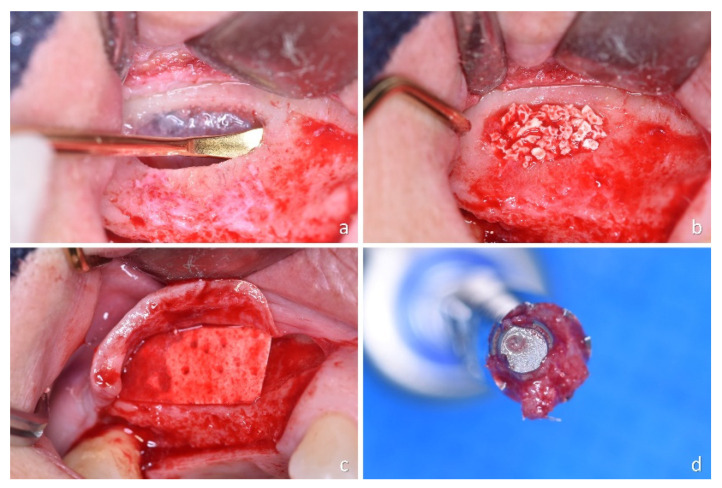
(**a**) Osteotomy and sinus mucosa elevation; (**b**) graft within the elevated space; (**c**) collagen membrane covering the access window; and (**d**) apical view of the biopsy: observe the eccentric position of the implant.

Two custom-made titanium screw-shaped mini-implants (Sweden & Martina, Due Carrare, Padua, Italy), 2.4 mm in diameter and 8 mm long, with either a moderately rough (ZirTi^®^ surface, Sweden & Martina, Due Carrare, Padua, Italy) [31] or a turned surface, were used (Figure 1).

Cerabone granulate 1.0–2.0 mm (Botiss Biomaterials GmbH, Zossen, Germany) was used as filler material. It is composed of a ceramic made of hydroxyapatite (pentacalcium hydroxide trisphosphate) obtained from bovine cancellous bone at a high-temperature (>1200 °C). It has macroporosities with a range of 100–1500 µm in dimensions.

The collagen membrane used to protect the access window was a Collprotect membrane (Botiss Biomaterials GmbH) obtained from porcine corium.

### 2.4. Sample Size

The sample size for the tomographic evaluations was reported in a previous article [30]. For the present article, the data from a previous study performed on dogs by the same group were used [18], and in which a statistically significant difference was obtained using 6 animals. A sample of 9 subjects in each group was calculated to be sufficient in a one-tail test to disclose differences between the two surfaces in bone-to-implant contact, with a power 0.8, an α error of 0.05, and an effect size of 0.96.

### 2.5. Study Design and Allocation Concealment

This was a triple-blind study because the participants, the surgeon and the assessor of the outcome were not informed about allocation treatment. The surgeon was informed after the preparation of the two osteotomies of the recipient sites. Two mini-implants were placed in the edentulous distal segment of the maxilla in the elevated region. The position (distal or mesial) was randomly allocated. The implants were installed by an expert surgeon (MF) while the randomization of the mini-implant position was performed by another author (DB). The randomization was performed electronically by an author not involved in the mini-implant installation and biopsy retrieval (DB). The treatment assignments were kept in opaque sealed envelopes that were opened after the preparation of the two osteotomies of the recipient sites.

### 2.6. Clinical Procedures

Detailed descriptions of the surgical procedures were reported in a previous article [30]. Briefly, lateral bone windows were prepared using a sonic-air surgical instrument (Sonosurgery^®^ TKD, Calenzano, FI, Italy), the sinus mucosa was elevated (Figure 1a), and a graft was used to fill the subantral space (Figure 1b). A collagen membrane was placed to cover the access window at the control sites (Figure 1c) while no membrane was used at the test sites. After 6 months of healing, two mini-implants were installed and retrieved after 3 months of submerged healing. A trephine (GA33M, Bontempi Strumenti Chirurgici, San Giovanni in Marignano, RN, Italy), 3.5 mm and 4 mm of internal and external diameter, respectively, was used, adopting an eccentric method to retrieve biopsies containing the mini-implants (Figure 1d) [32]. Standard implants were subsequently installed in the same position.

### 2.7. Histological Preparation of the Biopsies

The biopsies were not removed from the trephines to avoid damages and were immediately fixed in 10% buffered formalin, followed by dehydration in an ascending series of alcohol, inclusion in resin (Technovit^®^ 7200 VLC; Kulzer, Wehrheim, Germany), and polymerization. Histological slide of ~30 µm of width were prepared following the longitudinal axis of the mini-implant and stained with acid fuchsine and toluidine blue.

### 2.8. Histomorphometric Evaluation

The histomorphometric evaluation were performed by a well-trained author (KAAA) blinded about allocations of the two mini-implants and an intra-rate agreement K > 0.90 was achieved. High-definition scanned photomicrographs (×200) of each histological slide were taken at an Eclipse Ci microscope (Nikon Corporation, Tokyo, Japan) equipped with a motorized stage (EK14 Nikon Corporation, Tokyo, Japan). The software NIS-Elements D 5.11.01 (Laboratory Imaging, Nikon Corporation, Tokyo, Japan) was used for histomorphometric measurements.

All measurements were performed from the most coronal contact of the bone to the implant surface to the apex. New bone, pre-existing bone (old bone and bone particles), residual graft, interpenetrating bone network (IBN; new bone penetrating the biomaterial), soft tissues (bone marrow, vessels) in contact to the implant surface (histometric linear measurements) and within 400 µm from the implant surface (morphometric measurements) were assessed.

For the morphometric measurements, a point counting method was applied [33], using a lattice with squares of 50 microns.

### 2.9. Data Analysis

Mean values are reported within the text while mean values and standard deviations as well as the 25th, 50th (median), and 75th percentiles are illustrated in the tables. The primary variable was new bone for both linear and morphometric evaluations. The other variables were considered as secondary variable.

Prism 9.1.1 (GraphPad Software, LLC, San Diego, CA, USA) was used for statistical analyses. The Shapiro–Wilk test was used to verify the normal distribution and either a paired t test or a Wilcoxon test was used to evaluate differences between rough and turned surface groups while an unpaired t test or a Mann–Whitney test was used to analyze differences between collagen membrane and no membrane groups. The level of significance was set at α 0.05. Pooled data with relation to the surface characteristics were also evaluated.

## 3. Results

### 3.1. Clinical Outcomes

**Figure 2:**
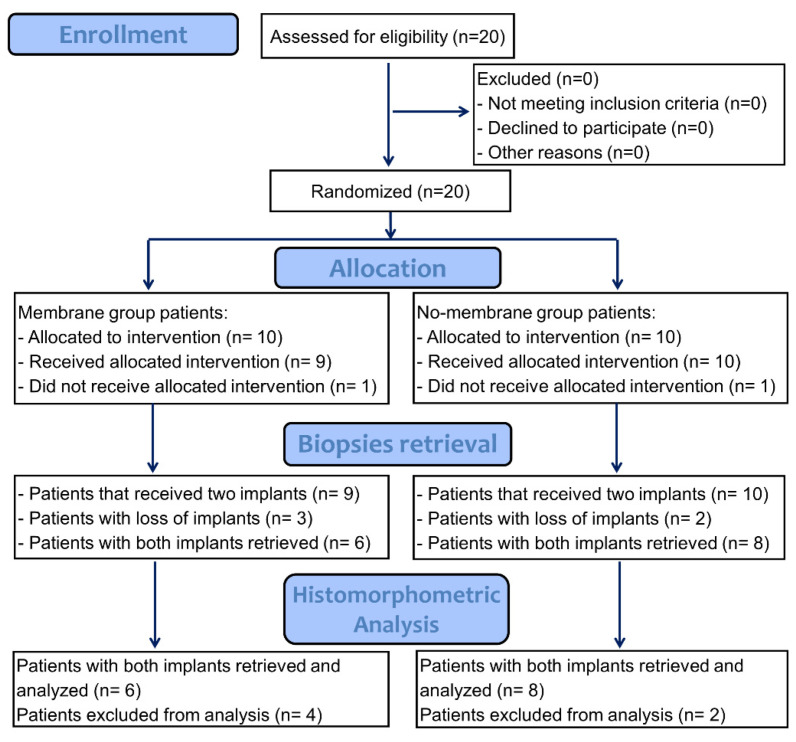
Consort flow diagram.

Twenty patients were initially included in the study. Two sinus mucosa perforations, one in each group, occurred during the surgical procedures. Both were protected with a collagen membrane. No complications were reported or observed during the healing period. Further clinical and radiographic information were reported elsewhere [30]. After 6 months, in one patient of the membrane group, insufficient hard tissue was found to install both mini-implants so that the patient was excluded from the histological analysis. After a further 3 months, at the time of biopsies removal, in five patients the mini-implants were not integrated. Hence, both mini-implants were finally retrieved from fourteen patients, six patients for the membrane group (*n* = 6) and eight patients for the no-membrane group (Table 1; *n* = 8; Figure 2).

### 3.2. Histometric Evaluations—Tissues in Contact with the Implant Surface

**Figure 3:**
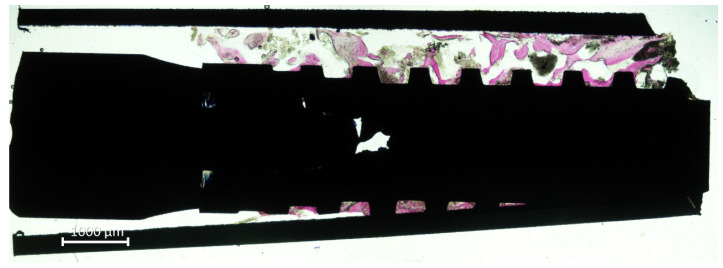
Retrieved biopsy. Note the eccentric position on the mini-implant within the trephine.

All biopsies were retrieved applying the eccentric method (Figure 3).

**Figure 4:**
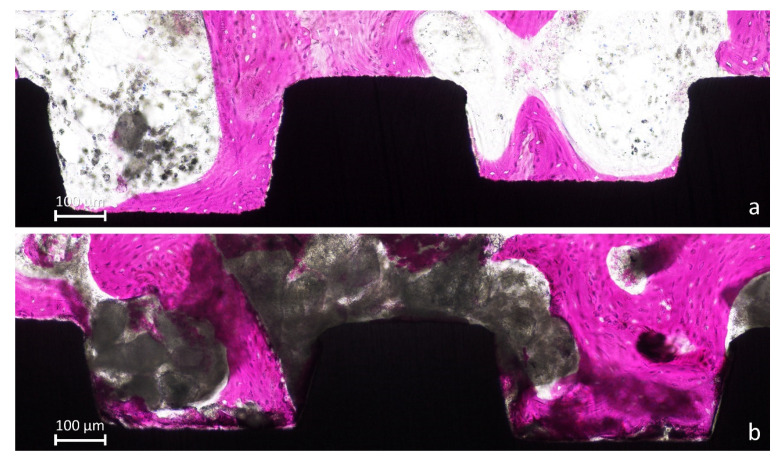
(**a**), New bone anchored to the implant surface. (**b**) Large amounts of biomaterial were still present.

The mini-implants presented new bone around and in contact to the surface (Figure 4a) while, in other regions, large amounts of biomaterial were still present (Figure 4b).

**Figure 5:**
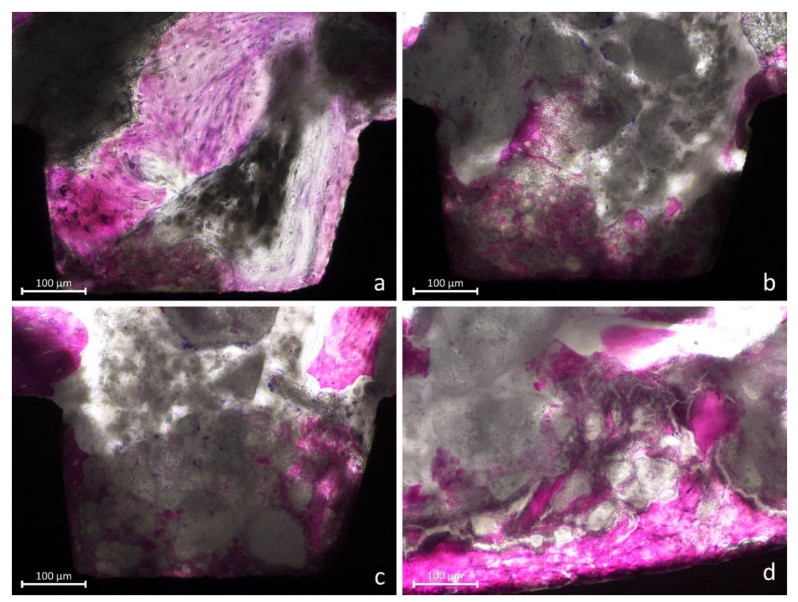
(**a**–**d**) Images showing new bone formed around and within the graft residues (interpenetrating bone network; IBN).

In several instances, the biomaterial was found overlaying the new bone, taking on a foggy appearance (Figure 5a–d). In such cases, that new bone was assuming a different feature compared to new bone outside the biomaterials, as if the two tissues were interpenetrating each other (interpenetrating bone network; IBN).

**Figure 6:**
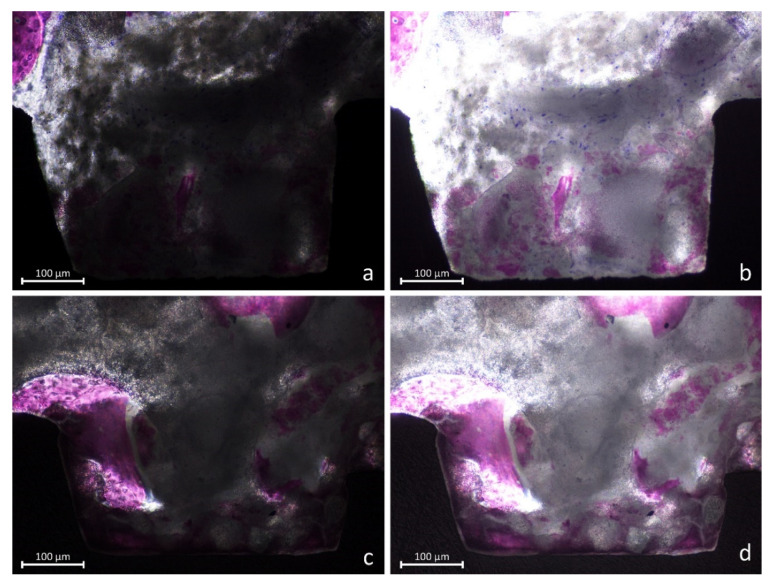
(**a**–**d**) Photomicrographs representing new bone and interpenetrating bone network (IBN). (**a**,**c**) Dark mode, at which a normal light exposure was adopted. (**b**,**d**) Overexposed images that better revealed the structure of the IBN.

High light intensity was provided to better identify this structure (Figure 6a–d).

**Figure 7:**
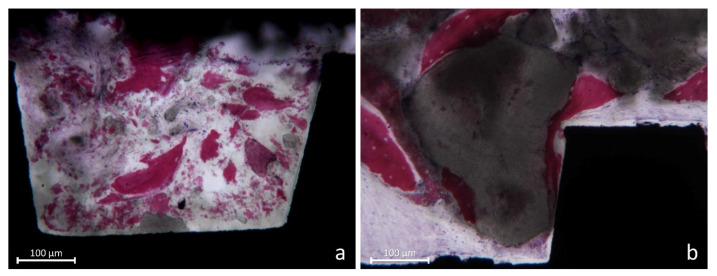
(**a**) Some bone particles not yet resorbed or included in new bone. (**b**) Granules of Cerabone^®^ surrounded by newly formed bone.

Bone particles were sometimes identified (Figure 7a) as well granules of biomaterial (Figure 7b).

**Figure 8:**
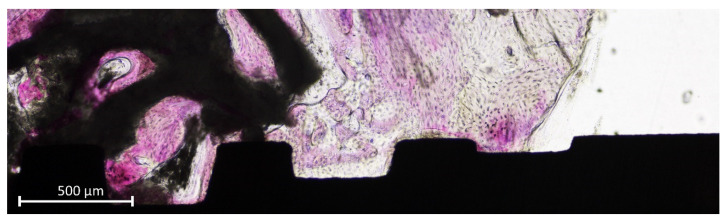
Old pre-existing bone at the coronal margin of the implant.

In the coronal segment of the mini-implant, old bone was still visible and was in some cases anchored to the implant surface (Figure 8).

The mean percentage of new bone in contact with the implant surface was higher at the ZirTi compared to the turned surfaces in both membrane (28.9% and 11.0%, respectively; *p* = 0.030; Table 2) and no-membrane groups (30.5% and 9.2%, respectively; *p* = 0.008; Table 3).

The difference between the membrane and no-membrane groups was not statistically significant for both ZirTi (*p* = 0.852) and turned (*p* = 0.636) surfaces.

The interpenetrating bone network (IBN) was in the membrane group 13.5% and 16.6% at the ZirTi and turned surfaces, respectively. In the no-membrane group, the respective fractions were 7.0% and 6.1%. In the membrane group, the sum between new bone and IBN yielded 42.4% of total bone for the ZirTi surface and 27.6 % for the turned surface (*p* = 0.258). In the no-membrane group, the respective percentages were 37.5% and 15.3 % (*p* = 0.001).

Small amounts of old bone (mean ≤ 3%) were observed while large remnants of non-resorbed graft were present in contact with the implant surface, the means ranging between 23.4% and 30.6%. Soft tissues were present in high percentages, ranging from 28.9% to 58.9%. The highest values were observed at the turned compared to the ZirTi surfaces. However, the difference was statistically significant only in the no-membrane group (*p* = 0.016).

The pooled data (Table 4) revealed that ZirTi surface yielded a higher amount of new bone (29.8%) and total bone (39.6%) compared to the turned surface (10% and 20.6%, respectively). Similar amounts of IBN, old bone and graft percentages were found at the two surfaces while statistically high percentages of soft tissues were detected at the turned compared to the ZirTi surfaces.

### 3.3. Morphometric Evaluations

A similar density of new bone was found around both ZirTi and Turned surfaces in both membrane and no-membrane groups, the means ranging between 19.9% and 23.7% (Table 5, Table 6 and Table 7). IBN means ranged between 11.4% to 6.3% and the total bone from 28.9% and 31.3%. Graft remnants were still present in a high proportion, ranging between 28.9% and 46.2%. No statistically significant differences were found for all variables above mentioned between surfaces and between membrane and no-membrane groups. Small amounts of old bone were detected while softs tissues ranged between 24.5% and 39.0%.

## 4. Discussion

The mini-implants retrieved were osseointegrated into newly formed bone. The different characteristics of the implant surface played an important role in osseointegration, generating a statistically significant higher amount of newly formed bone at the moderately rough compared to the turned surface. However, no differences could be detected between the membrane and no-membrane groups.

A total of ten mini-implants were found not integrated, independently from the surface characteristics. This is not in agreement with other RCTs that included a similar design with mini-implant installed after 6 months from sinus floor elevation and retrieved after a further 3 months [27,28]. In those studies, different dimensions and positions of the access window were included as variables, and a collagenated cortico-cancellous porcine bone was used as filler. Only implants with a moderate surface were used. Four mini-implants were lost in one study [27] while none in the other study [28].

Nevertheless, the xenogeneic graft used in the present study has been used in several studies that reported optimal results both in clinical [34,35,36,37,38] and animal [39,40,41,42] studies.

Even though in the present study the loss of implants was similar for both surfaces, the grade of osseointegration was statistically significantly higher at the moderately rough compared to the turned surface. It should be considered that, when an implant is installed in a standard alveolar crest, new bone can be formed from multiple sources, both in the cortical and marrow regions. A strong cellular reaction can be observed after 5 days of healing within the marrow compartment around the body of the implant [43]. New bone is subsequently formed, creating a bone barrier around the implant and on its surface showing an attempt to isolate the implant body from the marrow compartment. In the cortical region, the old pre-existing marginal bone around the implant is resorbed over time and substituted by newly formed bone, mainly through basic multicellular units (BMUs) [43]. Under such conditions of multiple bone sources, also a turned surface might work properly. Indeed, in an experiment in dogs, both surfaces were integrated 4 months after the installation in a healed alveolar bone [18] presenting osseointegration fractions of 56.3% and 50.6% at the moderately rough and turned surfaces, respectively. However, the presence of residual graft particles in composite bone limits the number of multiple bone sources [27,28], and in such a case, the degree of osteoconductivity of the implant surface might play an important role.

The importance of osteoconductivity properties has been elucidated in an experiment in dogs [44]. In that study, circumferential marginal defects with a depth of 5 mm and a horizontal gap of 1.25 were created around implants presenting either a moderately rough or a turned surface. Collagen membranes were used to protect the defects. Both submerged and not submerged healing were studied. After 4 months of healing, the marginal gain at the moderately rough surface was >4 mm while, at the turned surface, residual defects of about 3.4–3.6 mm in depth were still present at both submerged and not submerged implants. Residual defects were also observed in another study in which commercial turned implants were used [45]. Marginal defects, 5 mm in depth but with horizontal gaps of different dimensions, were tested. It was shown that the larger the marginal defect at installation, the deeper the residual defect after 12 weeks of healing. Moreover, it was shown that, due to the small horizontal dimensions, the residual marginal defects were not detectable at a clinical evaluation.

In other experimental studies, only moderately rough surfaces were adopted, and marginal defects were prepared. It was shown that, in the presence of marginal defects, the new bone was formed from the lateral bone walls during the first month of healing and the lateral growth stopped at ~0.4 mm from the implant surface [46,47]. During the same period, osseointegration started from the base of the defect and proceeded coronally to gain the closure of the defect in few months [46,48]. This period of healing is longer compared to that needed for the healing of artificial defects and extraction sockets [49,50,51,52]. Similar marginal defects, but larger in dimensions compared to those artificial described above, are obtained at implants installed simultaneously to sinus floor elevation performed by lateral or transcrestal accesses. In that case, new bone apposition on the implant surface starts from the sinus floor proceeding towards the apex [12,53], and reaches the implant apex, but only if the conditions for the growth are maintained over time [54].

However, in the present study, the mini-implants were inserted 6 months after sinus floor elevation. It might be argued that bone regeneration in that area should have already created similar conditions to that of a pristine alveolar bone. However, high amounts of residual grafts were still present after 9 months from the first surgery, providing different characteristics to the regenerated grafted bone (composite bone) compared to the pristine alveolar bone. Like in the present study, other similar RCTs showed a contact of the graft to the surface at implants installed after 6 months from sinus floor elevation and retrieved after 3 more months [27,28]. The histological analyses revealed 0.6–15.9% of graft in contact to the implant surface. This contact of the biomaterial to the surface reduced the available space for bone apposition as well as the number of bone sources compared to a pristine alveolar bone. In addition, it has to be considered that bone density was similar around both moderately rough and turned surfaces so that bone sources availability should be considered similar for the two different surfaces. Under such conditions, the osteoconductivity of the surfaces acquires an important role and the turned surface might be at a disadvantage compared to the moderately rough surface. This condition resulted in a much lower BIC% at the former compared to the latter surface than it was expected [18]. In fact, in the present study, the difference in pooled BIC% between the two groups of surfaces was ~19% while the difference found in another study between two similar surfaces at implants installed in pristine bone was 5.7% [18].

In the present study, the term “interpenetrating bone network (IBN)” was used. This term was first used in an experimental study in which a biphasic biomaterial, composed of 60% hydroxyapatite (HA) and 40% of beta-tricalcium phosphate (β-TCP), was used as filler material for sinus augmentation in rabbits [55]. Providing a higher light intensity at the optical microscope, it was possible to identify new bone overlapping or within the graft residues (Figure 6a–d). This was shown also in another previously published article on sinus floor elevation in sheep in which a biphasic biomaterial was also used as filler, again composed of HA 60% and β-TCP 40% [56]. The structure of the IBN recalled the structure of an “interpenetrating polymer network” [57] and, for this reason, the term “interpenetrating bone network” was adopted.

The foggy-like appearance of the biomaterial and the presence of old bone particles might depose for damage of the newly formed composite bone within the elevated area that occurred during the recipient site preparation for implant installation. Moreover, the cellular reaction that follows this event [45] triggered new bone formation and further degradation of the biomaterial.

No statistically significant differences were seen for osseointegration of mini-implants and bone density between membrane and no-membrane groups for both surfaces evaluated. Nevertheless, a systematic review concluded that better results might be obtained in implant survival after 3 years using a rough surface and a membrane coverage of the access window [1]. The results from the present study agree with the former but disagree with the second assumption. It has to be considered that, even though histological studies reported higher amounts of new bone density using membrane either in PTFE [58] or collagen [59,60,61], in those studies the biopsies were taken through the access windows so that the data does not represent correctly the region of interest. Data supporting this assumption were reported in a histological study in humans in which biopsies were taken after 9 months after sinus floor elevation [62]. Statistically higher fractions of mineralized bone were found at the biopsies taken from the alveolar crest (40.1%) compared to those taken from the lateral window (26.0%), even though the osteotomy was protected with a collagen membrane at the time of sinus floor elevation. Moreover, in a systematic review with meta-analysis [17], it was concluded that a membrane placed on the access window does not influence the proportion of bone formed within the elevated space. This outcome was also supported by the data from another experimental study on sinus floor augmentation in rabbits in which the percentages of new bone within the grafted sinuses after 8 weeks of healing were 24.9% and 24.5% for membrane and no-membrane groups, respectively [63].

The main limitation of the present study is related to the low numbers of patients included and of the biopsies retrieved. Nevertheless, the importance of the implant surface osteoconductivity has been clearly shown. Another limitation is the biomaterial used that might have influenced bone formation within the sinus cavity so that the results should not be inferred with other fillers. Studies comparing the present with biomaterial devoid of a similar property of interpenetration should be performed. RCTs using moderate rough and turned surfaces should be performed to compare the healing at implants installed in the pristine or composite alveolar bone. The results from the present study suggest that the osteoconductivity properties of the surface should be considered when the implant is installed in the composite bone because the residual graft might interfere with the osseointegration processes. In the present study, a bovine cancellous bone and a porcine corium collagen membrane were used as biomaterial. Other biomaterials that proved their capacity of supporting tissue regeneration should be used and evaluated [64,65,66,67].

The presence of graft material in contact with the implant surface suggests that composite bone might result in critical regions in which no integration of the implant surface occurs. This condition suggests the need of further investigations to identify the best biomaterials able to reduce this phenomenon and the capacity of the implant surface to favorite bone apposition also in the presence of composite bone.

## 5. Conclusions

It might be concluded that implants with a moderately rough surface installed in a composite bone presented much higher osseointegration compared to those with a turned surface. The present study failed to show the effect of the use of a collagen membrane on the access window.

## Figures and Tables

**Table 1:** Demographic data.

	Number	Age	Smokers	Mb	No-Mb
Females	10	53.1 ± 9.3	10 No	5	5
Males	4	59.0 ± 12.8	4 No	3	1

**Table 2:** Membrane group (*n* = 6). Tissues in contact to the implant surface expressed in percentages (%). SD, standard deviation. IBN, interpenetrating bone network; 25%, first percentile; 75%, third percentile.

		New Bone	IBN	Total Bone	Old Bone	Graft	Soft Tissues
ZIRTI	Mean ± SD Median (25%; 75%)	28.9 ± 14.5 25.2 (24.3; 34.1)	13.5 ± 8.0 14.6 (7.7; 20.1)	42.4 ± 17.7 35.5 (30.3; 48.1)	1.6 ± 3.8 0.0 (0.0; 0.0)	25.2 ± 15.2 24.0 (15.7; 29.7)	30.8 ± 17.3 37.3 (15.9; 43.3)
TURNED	Mean ± SD Median (25%; 75%)	11.0 ± 5.7 12.9 (8.0; 14.2)	16.6 ± 15.1 11.2 (5.7; 27.6)	27.6 ± 14.5 23.1 (17.0; 33.3)	1.2 ± 2.1 0.0 (0.0; 1.7)	27.2 ± 17.7 29.7 (15.7; 38.3)	43.9 ± 26.0 38.4 (34.2; 59.6)
*p*-value ZirTi vs. Turned		0.030	0.750	0.258	>0.999	0.828	0.305
*p*-value Mb vs. No-Mb ZirTi		0.852	0.108	0.612	0.469	0.579	0.507
*p*-value Mb vs. No-Mb Turned		0.636	0.308	0.103	0.618	0.755	0.282

**Table 3:** No-membrane group (*n* = 8). Tissues in contact to the implant surface expressed in percentages (%). SD, standard deviation. IBN, interpenetrating bone network; 25%, first percentile; 75%, third percentile.

		New Bone	IBN	Total Bone	Old Bone	Graft	Soft Tissues
ZIRTI	Mean ± SD Median (25%; 75%)	30.5 ± 14.9 27.1 (19.1; 34.7)	7.0 ± 8.1 3.8 (1.6; 9.0)	37.5 ± 17.3 32.4 (26.3; 43.5)	3.0 ± 3.6 1.3 (0.0; 5.5)	30.6 ± 20.6 35.6 (12.1; 46.0)	28.9 ± 12.6 27.9 (23.0; 30.1)
TURNED	Mean ± SD Median (25%; 75%)	9.2 ± 7.3 6.5 (5.1; 13.1)	6.1 ± 6.1 5.8 (0.5; 9.7)	15.3 ± 8.1 13.9 (8.7; 21.7)	2.4 ± 4.2 0.3 (0.0; 3.1)	23.4 ± 24.3 14.4 (10.6; 28.2)	58.9 ± 23.4 70.6 (47.9; 75.4)
*p*-value ZirTi vs. Turned		0.008	0.672	0.001	0.625	0.461	0.016

**Table 4:** Pooled data of membrane and no-membrane groups (*n* = 14). Tissues in contact to the implant surface expressed in percentages (%). SD, standard deviation. IBN, interpenetrating bone network; 25%, first percentile; 75%, third percentile.

		New Bone	IBN	Total Bone	Old Bone	Graft	Soft Tissues
ZIRTI	Mean ± SD Median (25%; 75%)	29.8 ± 14.2 26.0 (20.9; 34.9)	9.8 ± 8.4 5.9 (2.4; 16.7)	39.6 ± 17.0 32.9 (28.9; 48.1)	2.4 ± 3.7 0.0 (0.0; 4.2)	28.3 ± 18.1 28.5 (13.4; 43.9)	29.7 ± 14.2 29.0 (19.4; 38.4)
TURNED	Mean ± SD Median (25%; 75%)	10.0 ± 6.5 9.2 (5.4; 14.2)	10.6 ± 11.7 9.3 (2.3; 11.5)	20.6 ± 12.5 17.1 (12.5; 27.0)	1.9 ± 3.4 0.0 (0.0; 2.7)	25.0 ± 21.0 17.5 (12.0; 38.3)	52.5 ± 24.8 58.7 (37.6; 74.5)
*p*-value ZirTi vs. Turned		0.000	0.594	0.003	0.813	0.580	0.004

**Table 5:** Membrane group (*n* = 6). Tissues density around the implant surface expressed in percentages (%). SD, standard deviation. IBN, interpenetrating bone network; 25%, first percentile; 75%, third percentile.

		New Bone	IBN	Total Bone	Old Bone	Graft	Soft Tissues
ZIRTI	Mean ± SD Median (25%; 75%)	21.8 ± 4.8 22.9 (18.1; 23.7)	7.4 ± 4.0 6.6 (5.0; 8.5)	29.2 ± 7.0 27.3 (25.2; 28.2)	0.1 ± 0.2 0.0 (0.0; 0.0)	46.2 ± 4.6 45.3 (42.9; 50.1)	24.5 ± 7.5 26.9 (21.2; 30.4)
TURNED	Mean ± SD Median (25%; 75%)	19.9 ± 8.9 21.2 (18.5; 22.0)	11.4 ± 9.0 8.4 (6.6; 11.2)	31.3 ± 6.2 31.6 (28.9; 33.7)	3.2 ± 4.5 0.7 (0.0; 5.5)	36.6 ± 11.3 39.5 (34.5; 43.5)	28.9 ± 5.4 31.0 (24.8; 32.7)
*p*-value ZirTi vs. Turned		0.552	0.438	0.563	0.250	0.048	0.282
*p*-value Mb vs. No-Mb ZirTi		0.662	0.878	>0.9999	0.021	0.342	0.883
*p*-value Mb vs. No-Mb Turned		0.573	0.282	0.534	0.505	0.308	0.037

**Table 6:** No-membrane group (*n* = 8). Tissues density around the implant surface expressed in percentages (%). SD, standard deviation. IBN, interpenetrating bone network; 25%, first percentile; 75%, third percentile.

		New Bone	IBN	Total Bone	Old Bone	Graft	Soft Tissues
ZIRTI	Mean ± SD Median (25%; 75%)	23.7 ± 10.3 20.3 (17.1; 29.3)	7.1 ± 3.9 6.7 (3.4; 11.0)	30.7 ± 10.0 28.1 (22.7; 37.5)	4.9 ± 6.3 2.7 (0.4; 6.7)	39.1 ± 19.4 44.3 (25.7; 51.4)	25.4 ± 13.6 27.5 (12.5; 31.4)
TURNED	Mean ± SD Median (25%; 75%)	22.6 ± 8.2 22.1 (19.3; 25.9)	6.3 ± 3.2 6.2 (5.4; 7.4)	28.9 ± 7.6 31.3 (25.3; 33.6)	3.2 ± 3.7 2.2 (0.7; 3.9)	28.9 ± 15.7 26.2 (19.7; 32.7)	39.0 ± 10.3 41.1 (36.3; 44.9)
*p*-value ZirTi vs. Turned		0.771	0.558	0.550	0.375	0.318	0.121

**Table 7:** Pooled data of membrane and no-membrane groups (*n* = 14). Tissues density around the implant surface expressed in percentages (%). SD, standard deviation. IBN, interpenetrating bone network; 25%, first percentile; 75%, third percentile.

		New Bone	IBN	Total Bone	Old Bone	Graft	Soft Tissues
ZIRTI	Mean ± SD Median (25%; 75%)	22.9 ± 8.1 22.5 (17.0; 27.4)	7.2 ± 3.8 6.7 (3.9; 10.4)	30.1 ± 8.6 27.6 (24.4; 34.1)	2.8 ± 5.2 0.2 (0.0; 3.4)	42.1 ± 15.0 45.3 (41.2; 51.2)	25.0 ±11.0 27.5 (14.8; 30.6)
TURNED	Mean ± SD Median (25%; 75%)	21.5 ± 8.3 21.3 (18.5; 23.9)	8.5 ± 6.6 6.5 (5.8; 9.6)	29.9 ± 6.9 31.3 (27.8; 33.7)	3.2 ± 3.9 1.4 (0.1; 4.9)	32.2 ± 14.1 32.2 (21.1; 39.6)	34.7 ± 9.8 33.6 (27.1; 41.6)
*p*-value ZirTi vs. Turned		0.547	0.726	0.987	0.846	0.092	0.061

## Data Availability

Data are available on reasonable request.
